# Immunotherapy Reduces Allergen-Mediated CD66b Expression and Myeloperoxidase Levels on Human Neutrophils from Allergic Patients

**DOI:** 10.1371/journal.pone.0094558

**Published:** 2014-04-16

**Authors:** Rocio Aroca, Cristina Chamorro, Antonio Vega, Inmaculada Ventura, Elisa Gómez, Ramón Pérez-Cano, Miguel Blanca, Javier Monteseirín

**Affiliations:** 1 Servicio Regional de Inmunología y Alergia, Hospital Universitario Virgen Macarena, Sevilla, Spain; 2 Departamento de Medicina, Facultad de Medicina, Universidad de Sevilla, Sevilla, Spain; 3 Research Laboratory, Carlos Haya Hospital-Fundacion IMABIS, Málaga, Spain; Centre de Recherche Public de la Santé (CRP-Santé), Luxembourg

## Abstract

CD66b is a member of the carcinoembryonic antigen family, which mediates the adhesion between neutrophils and to endothelial cells. Allergen-specific immunotherapy is widely used to treat allergic diseases, and the molecular mechanisms underlying this therapy are poorly understood. The present work was undertaken to analyze A) the *in vitro* effect of allergens and immunotherapy on cell-surface CD66b expression of neutrophils from patients with allergic asthma and rhinitis and B) the *in vivo* effect of immunotherapy on cell-surface CD66b expression of neutrophils from nasal lavage fluid during the spring season. Myeloperoxidase expression and activity was also analyzed in nasal lavage fluid as a general marker of neutrophil activation.

**Results:**

CD66b cell-surface expression is upregulated *in vitro* in response to allergens, and significantly reduced by immunotherapy (p<0.001). Myeloperoxidase activity in nasal lavage fluid was also significantly reduced by immunotherapy, as were the neutrophil cell-surface expression of CD66b and myeloperoxidase (p<0.001). Interestingly, CD66b expression was higher in neutrophils from nasal lavage fluid than those from peripheral blood, and immunotherapy reduced the number of CD66^+^MPO^+^ cells in nasal lavage fluid. Thus, immunotherapy positive effects might, at least in part, be mediated by the negative regulation of the CD66b and myeloperoxidase activity in human neutrophils.

## Introduction

Four members of the CD66 antigens have been identified in human neutrophils, including CD66a, CD66d, CD66b, CD66c [1], [2]. The adhesion of neutrophils to the E-selectin at the endothelium is mediated by the interaction of this molecule and CD15, a E-selectin ligand which is present in the CD66 molecule [3]. Activation through CD66 antigens induces β_2_ integrin-mediated adhesion of neutrophils to fibrinogen [4]. The transmigration process of peripheral blood neutrophils is accompanied by highly restricted up- and down-regulation process of surface molecules. The resulting activation phenotype is characterized by increased integrin levels but also, by an extended expression of the surface molecule CD66b. In this context, CD66b is a well-known cell marker of activation [5], and of exocytosis of specific granules [6], but the functions of this molecule are largely unknown. Myeloperoxidase (MPO) is the most abundant proinflammatory enzyme stored in the azurophilic granules of neutrophilic granulocytes, accounting for approximately 5% of their dry mass [7]. MPO has been found to be implicated in allergic diseases (Reviewed in [8]).

Allergen-specific immunotherapy (IT) is the practice of administering allergen (Ags) extracts to patients with allergic disorders, in order to modify or abolish the clinical manifestations caused by natural exposure to the Ags. During the last 25 years, there has been an impressive development of basic and clinical research in the field of IT, and recent clinical trials have confirmed its effectiveness when properly indicated and conducted. IT has been validated as a causal treatment for respiratory allergy (rhinitis and asthma) and venom allergy [9], [10]. In children, it prevents new sensitizations [11] and the progression from rhinitis to asthma [12]. However, IT downmodulates the immune response by mechanisms that are not yet fully understood.

Previous studies have shown the presence of the three forms of IgE receptors on human neutrophils: FcεRI, FcεRII/CD23 and galectin-3 (reviewed in [8]). We have previously shown that specific Ags were able to activate functional responses by neutrophils from allergic patients sensitized to those Ags (reviewed in [8]). Thus, the present work was undertaken to analyze a possible implication of the CD66b molecule in allergic-mediated processes and the effect of IT.

## Materials and Methods

### Ethics statement

The Hospital Universitario Virgen Macarena ethics committee approved the study and each subject gave written informed consent (Ref: C.I. 1772).

### Materials

The Ags were commercially available antigen extracts, including D_1_ (*Dermatophagoides pteronyssinus*), G_3_ (*Dactylis Glomerata*), T_9_ (*olea europea*), and W_6_ (*Artemisia vulgaris*). They were purchased from Bial-Arístegui (Bilbao, Spain). Fluorescein isothiocyanate (FITC)-conjugated mouse monoclonal antibody (Ab) against human CD66b, and FITC-conjugated IgG isotype control were from Coulter-Izasa (Barcelona, Spain). Phycoerythrin (PE)-conjugated monoclonal Ab against human myeloperoxydase and PE-conjugated IgG isotype control were from Acris Antibodies (Hiddenhausen, Germany). *Escherichia coli* Lipopolysaccharide (LPS) was from Sigma-Aldrich (Madrid, Spain). Ficoll-Hypaque, Phosphate-buffered saline (PBS), RPMI 1640, fetal bovine serum, and Penicillin/Streptomycin were from Bio-Whittaker (Verviers, Belgium). All culture reagents used in this work (including Ags) had endotoxin levels of ≤0.01 ng/ml, as verified by the Coatest *Limulus* lysate assay (Chromogenix, Mölndal, Sweden).

### Patients and controls

Three groups were examined and compared: IT-treated adult atopic patients with allergic asthma and rhinitis (n = 15), non IT-treated adult atopic patients with allergic asthma and rhinitis (n = 10), and healthy non-atopic volunteer controls (n = 10) (summarized in Table 1). Intermittent bronchial asthma was diagnosed on the basis of criteria previously described in detail [13] and were randomly included in their respective groups (with or without IT). All subjects were lifelong non-smokers. The patients had positive skin-prick test (SPT) results (Bial-Arístegui) and serum specific-IgE (HYTEC 288, Hycor Biomedical-IZASA) to G_3_. The sensitivity of the assay was of 0.24 ng/ml to G_3_. G_3_ was choosen as the Ag of study due to the high frequency of sensitization to this allergen among the population of Seville, and the high prevalence of symptoms over other Ags in spring [14],[15]. The IT group received G_3_-immunotherapy (Bial-Aristegui) for the previous three years and continued to receive a maintenance dose within the highest dose of the extract. The healthy group had no history of allergy or bronchial symptoms, and had negative SPT results and demonstrated no specific-IgE responses to a battery of inhalant Ags (house-dust mites, pollens, moulds and animal danders). Patients were not allowed to take any bronchodilators within the 8 h before nasal challenge or challenge of cells *in vitro*. Oral bronchodilators were withheld for 24 h, and none of the donors had taken corticosteroids, disodium cromoglycate, nedocromil sodium or antihistamines in the previous week. None of the studied subjects had experienced respiratory-tract infections in the 4 weeks before blood sampling and/or nasal challenge.

**Table 1:** Demographic characteristics of the study groups.

Parameter	Healthy subjects (n = 10)	Non-IT treated patients (n = 10)	IT-treated patients (n = 15)
Age*	36,2±2,5	37,8±2,2	39,2±2,1
Age (max/min)	50/27	49/27	20/27
Gender (male/female)	5/5	4/6	8/7
G_3_-specific IgE*	0	55,2±12,2	16,8±5,7**
Score of symptoms*	0	100,5±35,5	27±7,9**

*Mean±SD.

**p<0.001, IT-treated group vs non-IT-treated group.

### Symptom scores

All patients recorded daily symptoms from eyes, nose and lungs: from the eyes itching, redness and tear flow, from the nose sneezing, nasal blockage and watery discharge, and from the lungs coughing, wheezing and trouble of breathing. Each symptom was graded from 0 to 3, where 0 represented no symptoms, 1 represented mild symptoms, 2 represented intermediate symptoms and 3 represented severe symptoms [16].

### Pollen count

Pollen counts were performed with the aid of a pollen trap (Burkard Seven Day Recording Volumetric Spore Trap Burkard Manufacturing Co. Ltd. Rickmansworth, Hertfordshire, UK) located on the roof (50 m above ground level) of the hospital building where the investigations were performed. The sucking rates were adjusted to 10 l/min, and the total number of pollen grains dispersed during a study week was calculated as the summation of the daily values (particles/m^3^ air) [17].

### Nasal lavage and myeloperoxydase activity analysis

A nasal lavage was performed when the grass pollen count was ≥1000 [17]. Nasal lavage fluids (NLFs) were obtained using a 30 ml syringe and the ‘nasal pool’ technique [17], [18]. After clearing excess mucus by forceful exsufflation, each nasal cavity was rinsed with 8 ml 0.9% NaCl solution, repeated five times. The suspension from both nasal cavities, ranging from 12 to 15 ml, was then centrifuged in 400×*g*, with slow acceleration and unbraked deceleration, at room temperature for 10 min. The supernatant was removed and 1 ml PBS solution with 10% fetal calf serum and 1% Varidase was added to the centrifuged cells.

Myeloperoxidase (MPO) activity was measured in the NLF by MPO-enzyme immunoassay (EIA) method (Oxis International, Portland, OR, USA) [15].

### Cell isolation and culture

Human neutrophils were isolated and culture as previously described (14). Briefly, neutrophil preparations were further purified using a MACS by incubation with mouse anti-human CD9, anti human CD203c, and anti-human CD14 Abs, and then with anti-mouse IgG micromagnetic beads. The purity of neutrophils was on average >99%. Neutrophils were cultured in RPMI 1640 medium supplemented with 10% (v/v) Fetal bovine serum, 2 mM L-glutamine, 100 U/ml penicillin, and100 µg/ml streptomycin, and maintained at 37°C in an atmosphere of 5% CO_2_ and 95% O_2_. For stimulation treatments, cells were incubated with Ags, anti-human Fcε receptors Abs or goat IgG, at 37°C for the times indicated. LPS was used at 1 µg/ml. None of the reagents affected the viability of the cells at the concentrations used in this work, as confirmed by the trypan blue dye-exclusion test.

### Dissociation of neutrophil-bound Igs

Ig molecules were dissociated from the cell-surface of neutrophils as described previously [19], [20], [21]. Briefly, after isolation, neutrophils were resuspended in 1 ml acetate buffer (50 mM sodium acetate (pH 4), 85 mM NaCl, 5 mM KCl, supplemented with 0.03% human serum albumin) and incubated on ice for 3 min. An equal volume of gelatin veronal buffer (1.8 mM sodium barbital, 3.1 mM barbituric acid, 0.1% gelatin, 0.05 mM MgCl_2_, 141 mM NaCl, 0.15 mM CaCl_2_ (pH 7.4)) was then added to the treated cells, and the mixture was centrifuged at 500×*g* for 10 min. After treatment, neutrophils were cultured with the different agents.

### Analysis of cell-surface CD66b and MPO by flow cytometry

Analysis of CD66b and MPO was performed as previously described [14]. Briefly, cells were rapidly cooled on ice, and 1 ml of PBS was added. Cells were then centrifuged at 500×*g* for 10 min, supernatants removed and cell pellets washed three times with ice-cold PBS before staining with 20 µl of FITC-conjugated mouse monoclonal Ab against human CD66b or PE-conjugated mouse monoclonal Ab against MPO. Isotype-matched mouse IgG served as control. Flow cytometry was performed on an Epics Elite flow cytometer (Coulter-Izasa, Barcelona, Spain) and calibrated by standard techniques. For each stained sample, 2×10^4^ cells were analyzed for fluorescence intensity. The fluorescence distribution for both isotype control and test cells was analyzed using a Coulter Elite software workstation. Results are expressed as the percentage of positive cells (%) or Mean Fluorescence Intensity (MFI).

### Statistics

Normality distribution was first examined using Shapiro-Wilk normality test before analysis of statistical significance. Data are expressed as means ± SD. A one-way ANOVA test was used to make comparisons between groups, except for the comparison of the score of symptoms and specific IgE levels in the IT and non-IT groups, where a Student *t* test was used instead. A level of p<0.05 was considered significant.

## Results

### IT decreases the levels of G_3_-specific IgE and the score of symptoms

Symptom scores and G_3_-specific IgE were both significantly lower (p<0.001) in patients of the IT-treated group than those from the non-IT-treated group (Table 1). These results confirm previous findings regarding the clinical effect of IT [22], [23].

### CD66b expression is upregulated from the cell-surface of human neutrophils in response to G_3_

**Figure 1:**
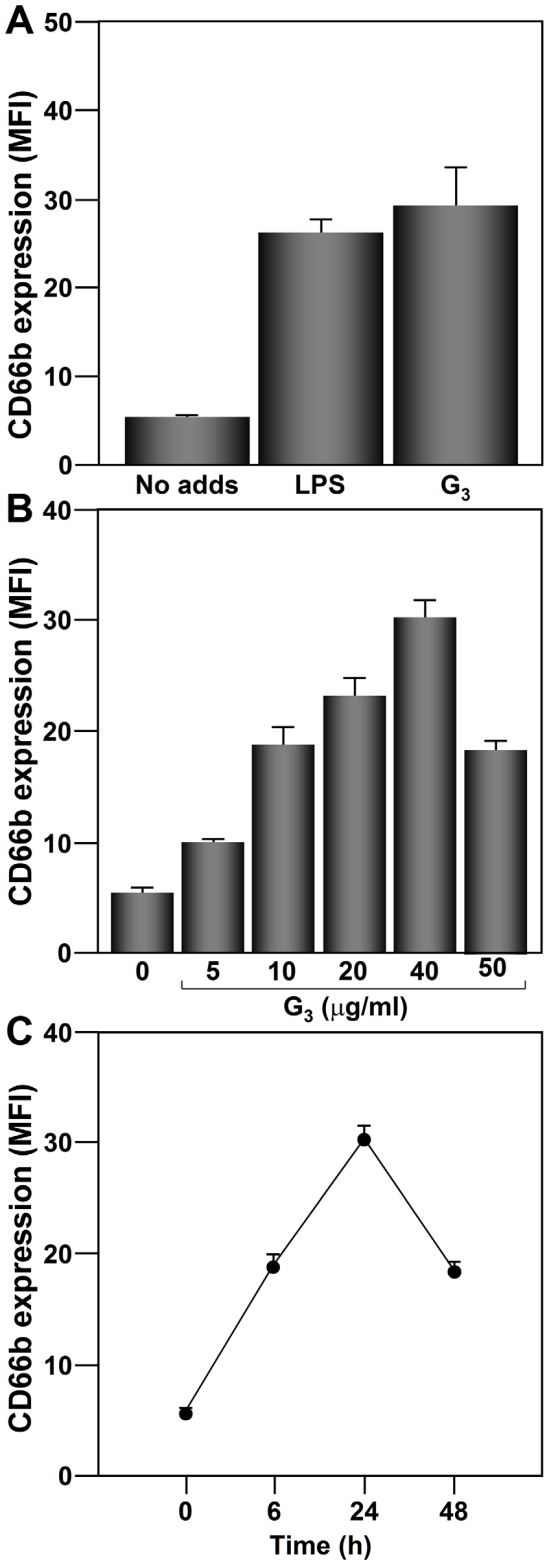
CD66b is upregulated from the cell-surface of neutrophils in response to G_3_. (**A**) Neutrophils (2×10^6^) from allergic patients (n = 10) were left untreated (No adds), treated with LPS (1 µg/ml) or an Ag (G_3_) to which the patient was sensitized (40 µg/ml) for 24 h. (**B**) and (**C**) Neutrophils from allergic patients (n = 10) were incubated with an Ag (G_3_) to which the patient was sensitized for the indicated doses (for 24 h) or times (at a dose of 40 µg/ml), and CD66b cell-surface expression was analyzed by flow cytometry. The results shown are the means ± SD.

Three types of IgE receptors/allergen-specific IgE molecules are present on human neutrophils (reviewed in [8]), prompting us to analyze whether challenging these cells with Ags was able to modulate the CD66b expression. Thus, neutrophils were cultured in the presence of an Ag to which the patients were sensitized (G_3_), or LPS as positive control [4], and the level of CD66b cell-surface expression was analyzed by flow cytometry. As is shown in Fig. 1A, the treatment with G_3_ or LPS induced an increase of CD66b expression as evidenced by the increase in the MFI (Fig. 1A). As shown in Fig. 1B and 1C, CD66b expression is upregulated in a dose- and time-dependent manner. CD66b expression upregulation was detected from 30 min, with a maximum level at 24 h, decreasing at 48 h. CD66b upregulation was detected at a G_3_ dose of 5 µg/ml, reaching the maximum at a dose of 40 µg/ml.

**Figure 2:**
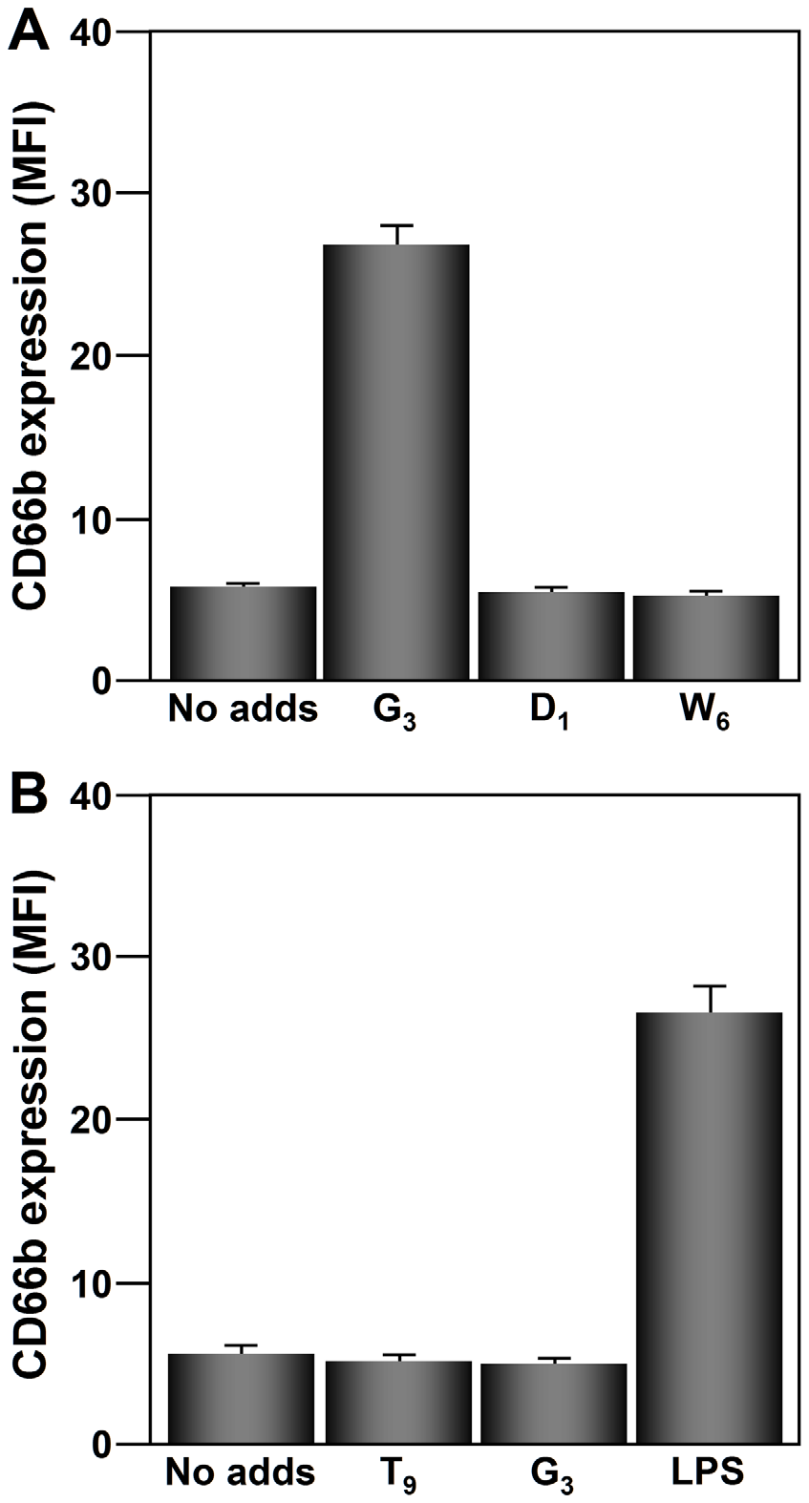
Specificity of the CD66b cell-surface expression upregulation in response to Ags. (**A**) Neutrophils (2×10^6^) from allergic patients (n = 10) sensitized to G_3_ but not to D_1_ and W_6_, were incubated with 40 µg/ml protein extracts of G_3_, D_1_, and W_6_ for 24 h. (**B**) Neutrophils from healthy subject (n = 10) were incubated with 40 µg/ml of T_9_, and G_3_ for 24 h. CD66b cell-surface expression was measured by flow cytometry. The values shown are the means ± SD.

Consistent with our previous observations (reviewed in [8]), the response of neutrophils to the Ag was specific. CD66b cell-surface expression is upregulated on neutrophils from allergic patients sensitized to *Dactylis glomerata* (G_3_) when cultured with this Ag (Fig. 2A). Other Ags to which the allergic patients were not sensitized (D_1_ and W_6_) did not affect CD66b expression (Fig. 2A). Neutrophils from healthy donors did not respond to any of the Ags tested, however they did respond to LPS (Fig. 2B).

**Figure 3:**
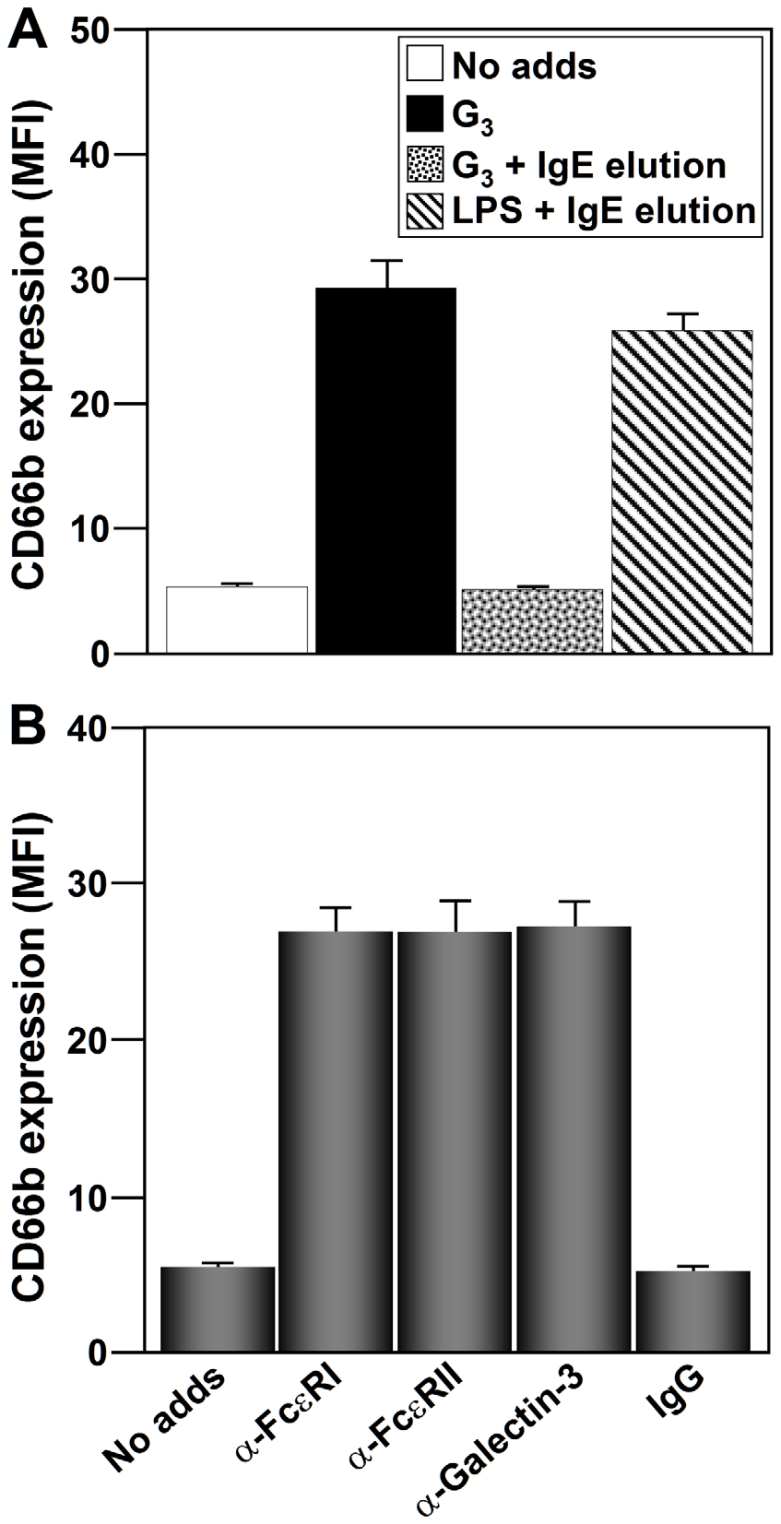
CD66 cell-surface expression upregulation in response to Ags is IgE-dependent. IgE receptors involved in the process. (**A**) Neutrophils (2×10^6^) from allergic patients sensitized to G_3_ (n = 10) were treated, where indicated, to elute the Igs from the cell surface, and then incubated with G_3_ (40 µg/ml) or LPS (1 µg/ml) for 24 h, and CD66 cell-surface expression was measured by flow cytometry. Specific IgE and IgG for the above Ags were determined in the elution solution by ELISA. (**B**) Neutrophils from allergic patients (n = 5) were incubated with anti-FcεRI (CRA 1), anti-CD23 (9P.25) or anti-galectin-3 (A3A12) antibodies at 5 µg/ml for 24 h, and CD66b cell-surface expression was measured as in A. The values shown are the means ± SD.

Only patients with positive SPT results and serum specific IgE to G_3_ showed specific IgE on the neutrophil surface as previously shown ([19] and data not shown). We could not detect specific IgG to the studied Ags on the neutrophil surface. Upon *in vitro* G_3_ challenge, CD66b was upregulated from only those neutrophils having cell-surface IgE specific to G_3_ (Fig. 3A). CD66b expression upregulation was not detected when IgE molecules were stripped from the neutrophil cell-surface before allergen challenge but was detected when neutrophils were incubated with LPS (Fig. 3A).

Experiments were thus performed to identify the main IgE receptor/s involved in Ag-dependent CD66b upregulation. The effect of agonist antibodies against each of the IgE receptors was examined as previously [19]. The antibodies used were: CRA1 (anti-FcεRI-chain), 9P.25 (anti-CD23/FcεRII) and A3A12 (anti-galectin-3/Mac-2). Antibodies against FcεRI, FcεRII, or Galectin-3 promoted similar CD66b upregulation (Fig. 3B). A non-specific mouse IgG antibody was used as a negative control (Fig. 3B).

### IT ameliorates the CD66b cell-surface upregulation induced by G_3_

**Figure 4:**
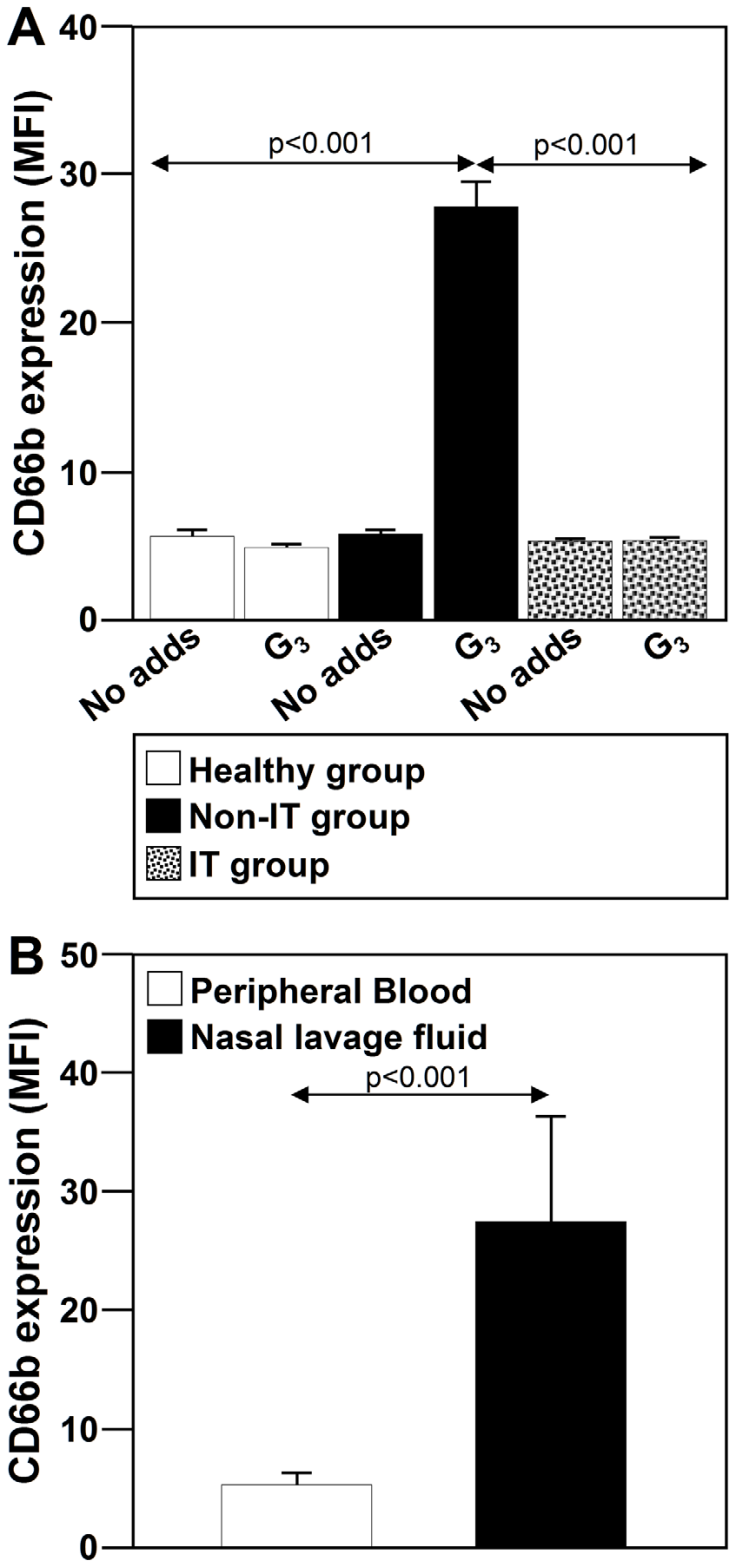
IT downmodulates the levels of CD66b cell-surface expression. CD66b expression is higher on neutrophils from NLP than those from peripheral blood. (**A**) Neutrophils (2×10^6^ cells) from healthy subjects (n = 10), non-IT-treated patients with allergic asthma and rhinitis sensitized to G_3_ (n = 10), and IT-treated patients with allergic asthma and rhinitis sensitized to G_3_ (n = 15) were left untreated (No adds) or treated with G_3_ (40 µg/ml) for 24 h. (**B**) Neutrophils purified from peripheral blood and NLF from non-IT treated patients. The levels of CD66b cell-surface expression were measured by flow cytometry. The values shown are the means ± SD.

Given that G_3_ which induce clinical symptoms induce CD66b cell-surface expression upregulation, we next asked whether this effect might be regulated by IT. As shown in Fig. 4A, IT decreased the CD66b expression upregulation induced by G_3_. The levels of CD66b upregulation were significantly lower on neutrophils from the group of IT-treated allergic patients than those from the group of non-IT-treated allergic patients (p<0.001) or the healthy donors group (p<0.001). No significant differences were observed between neutrophils from the group of healthy donors and of IT-treated allergic patients.

### CD66b cell-surface expression is higher on neutrophil from NLF than from peripheral blood, and IT reduces its expression

We next analyzed the levels of CD66b expression on neutrophils from NLF and peripheral blood of the non-IT-treated group. As shown in Fig. 4B, the expression of CD66b was higher on neutrophils from NLF than those from peripheral blood (p<0.001) (Fig. 4B).

**Figure 5:**
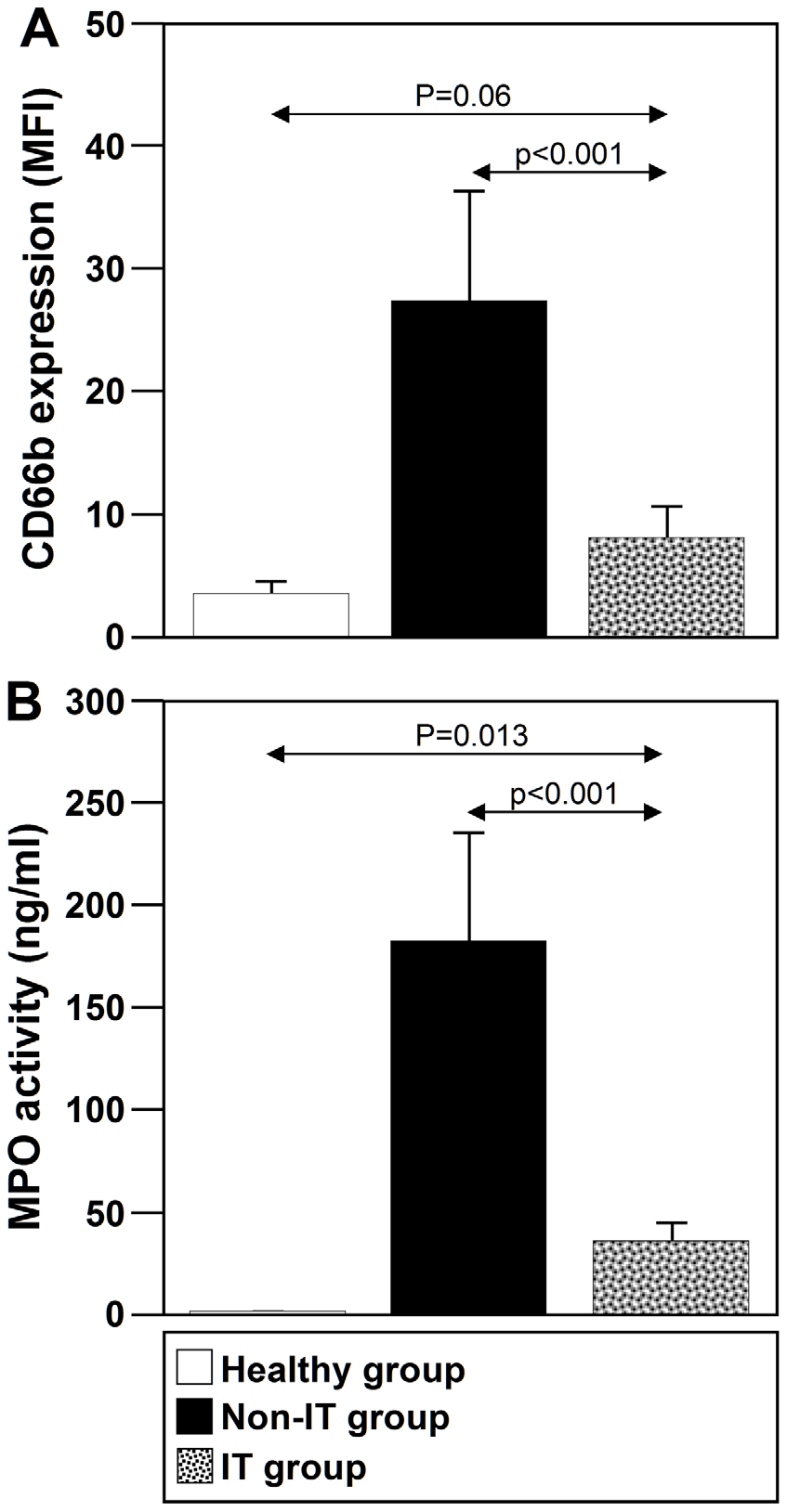
IT downmodulates the levels of CD66b cell-surface expression and MPO activity in NLF. (**A**) CD66b expression was analyzed on cells from NLF in healthy subjects (n = 10), IT-treated allergic patients (n = 15) and non-IT-treated allergic patients (n = 10). (**B**) MPO activity was analyzed in NLF from healthy subjects (n = 10), IT-treated allergic patients (n = 15) and non-IT-treated allergic patients (n = 10). The values shown are the mean ± SD.

Thus, we next analyzed the levels of CD66b expression on neutrophils from NLF of the three studied groups. As shown in Fig. 5A, the levels of cell-surface CD66b expression on neutrophils from healthy donors were similar to those from IT-treated patients (p = 0.06). However, CD66b levels were significantly lower on neutrophils from IT-treated patients than those from non-IT-treated patients (p<0.001).

### IT reduces MPO activity levels and the number of MPO^+^CD66b^+^ cells in NLF

To determine the effect of IT on neutrophil function, we analyzed MPO activity in the NLF from the three studied groups. MPO activity measured in NLF of the IT-treated group was significantly lower than that of the non-IT-treated group (p<0.001) but subtly higher than those of the healthy group (p = 0.013) (Fig. 5B).

**Figure 6:**
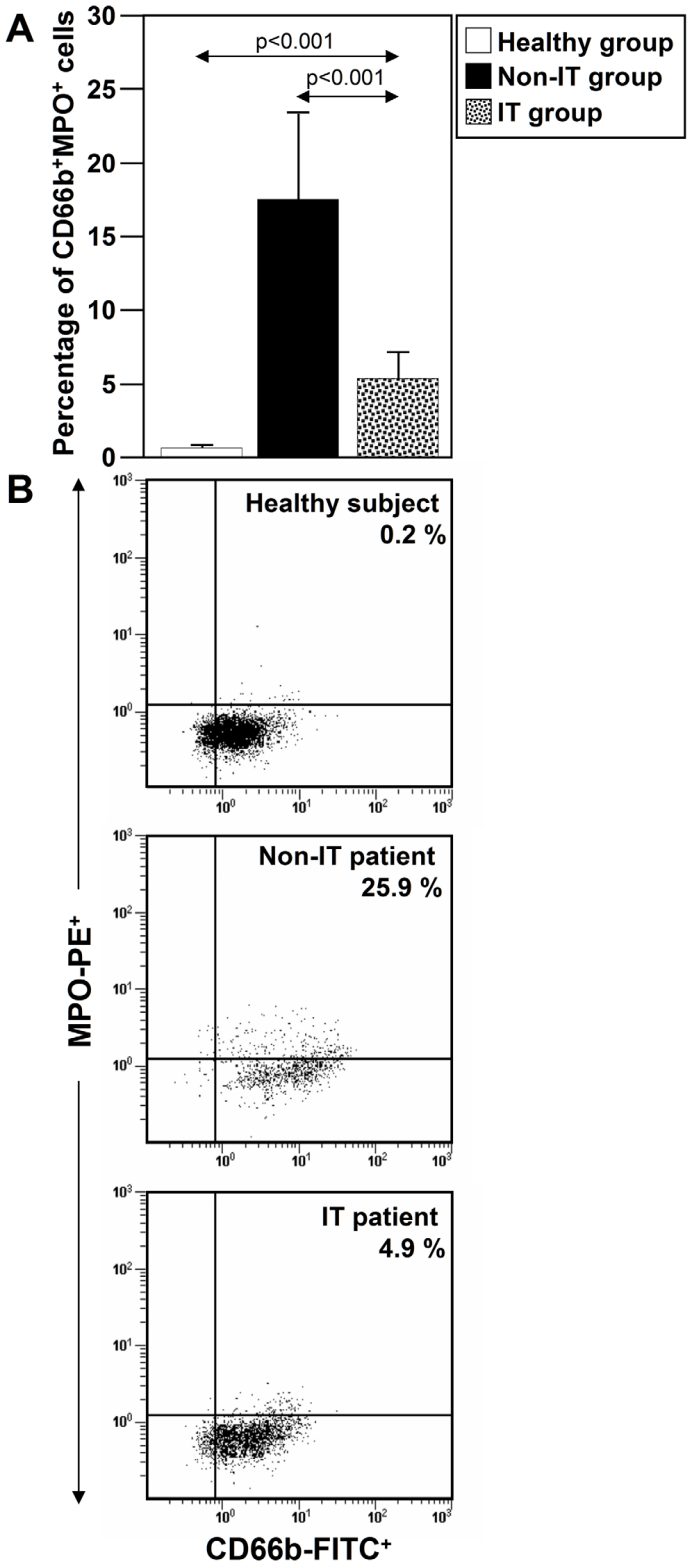
IT reduces the number of CD66b^+^MPO^+^ cells in NLF. (**A**) The percentage of CD66b^+^MPO^+^ cells was analyzed on cells from NLF in healthy subjects (n = 10), IT-treated allergic patients (n = 15) and non-IT-treated allergic patients (n = 10). The values shown are the mean ± SD. (**B**) Three representative dot-blot of the results shown in A.

Since cell surface associated-MPO is higher in neutrophils from allergic patients than those from healthy subjects [24], we used flow cytometry to measure MPO on neutrophils from NLF in the three studied groups. The number of MPO^+^CD66b^+^ cells in NLF of the IT-treated group was significantly lower than in the non-IT-treated group (p<0.001) but slightly higher than in the healthy group (p = 0.001) (Fig. 6A and 6B).

## Discussion

IT is an effective means of reorienting inappropriate immune responses in allergic patients. Administration of IT is a clinically efficient treatment for grass pollen–induced rhinoconjunctivitis and asthma, suggesting disease modification. In our study, the levels of score of symptoms and G_3_-specific IgE were significantly lower in patients of the IT-treated group than those of the non-IT-treated group. Thus, our results confirm previous findings regarding the clinical effect of IT [22], [23].

The results presented here provide evidence of a possible involvement of the CD66b molecule in the allergic asthma and rhinitis processes and reveal that IT has a positive effect on this molecule. Several lines of evidence verify this claim: 1) CD66b cell-surface expression is upregulated in neutrophils from patients with allergic asthma and rhinitis in response to only the Ag which induce clinical symptoms, 2) Ags did not affect CD66b cell-surface expression in on human neutrophils from healthy subjects or from IT-treated patients with allergic asthma and rhinitis, and 3) The expression of CD66b on neutrophils from NLF was higher than those from peripheral blood.

Normally, circulating neutrophils do not adhere to the vascular endothelium, but in inflammatory lesions the sequential engagement of a series of adhesion receptors leads to a multistep program to allow the activated cells to roll along the endothelium, firmly adhere, and then transmigrate across the endothelial wall. The initial contact between neutrophils and endothelial cells is mediated by L-selectin (CD62L) on neutrophils and leads to tethering and rolling of the neutrophils. The importance of L-selectin-mediated contact in the initiation of neutrophils rolling under shear flow conditions has been shown in numerous *in vitro* and *in vivo* models [8], [14]. Firm adhesion is then dependent on β2 integrins (CD11a/CD18 (LFA-1) and CD11b/CD18 (CR3, Mac-1)), which bind to ICAM-1 (CD54) on activated endothelial cells [8]. Up-regulation of CD11b/CD18 on neutrophils, however, is not sufficient to promote firm adhesion [8]. Expression of an activated form of CD11b/CD18 is required for firm adhesion [8]. Finally, CD31 (PECAM1), expressed on endothelial cells and neutrophils, is involved in the transmigration of leukocytes across the endothelial cell layer [8]. Other neutrophils surface proteins can also influence the adhesion process. CD66b interacts with E-selectin on endothelial cells and may activate CD11b/CD18 [3]. CD66b molecules seem to be capable of mediating a rapid and very effective cell adhesion, probably over a cross-linking-induced signal transduction. Brown et al. described that anti-CD11b antibodies are not very effective at inhibiting the interaction of neutrophils from patients with sepsis to endothelial monolayers. CD66b might be one candidate that contributes to the supranormal adhesiveness to the endothelium [25], [26].

MPO may alter intracellular signaling pathways in neutrophils upon adhering to integrins on the neutrophil membrane. MPO binds to CD11b/CD18 integrins on neutrophils, leading to induction of intracellular signaling cascades and translating into up-regulated neutrophils degranulation, CD11b surface expression, and NADPH oxidase activity in an autocrine manner. These properties of MPO add to the growing body of evidence characterizing MPO as a proinflammatory mediator and reveals alternative functions of this enzyme, which are irrespective of its bactericidal and enzymatic activity. Inflammation may be a localized phenomenon with predilection for specific sites. MPO was identified as a leucocyte activation marker specifically expressed at sites of inflammation in the arterial tree MPO is able to attract neutrophils towards the vascular wall, thereby inducing inflammation [24], [27].

We have previously shown that on neutrophils from allergic patients the expression of CD11b/CD18 and CD62L is, respectively, upregulated and downmodulated in an IgE-dependent manner [8], [14]. Neutrophils expressing higher levels of adhesion molecules would preferentially migrate to the lungs and nose following allergic challenge, and therefore the cells remaining would consist of a population with lower surface levels of adhesion molecules. These data suggest that these adhesion molecules are key to neutrophil recruitment to the lungs and nose in allergic processes [17], [28], [29], [30], [31]. In addition to the presented data, we have previously shown that the activation of neutrophils from allergic patient through the IgE-receptor galectin-3 induces the downmodulation of CD62L expression and the release of key inflammatory mediators [8], [14]. In this sense, CD66b has also been shown to be the functional galectin-3 receptor [32], this suggesting that there is a complex adhesion molecules profile involved in allergic inflammatory processes.

IT downmodulated the CD66b cell-surface expression upregulation induced by G_3_ *in vitro* on human neutrophils from patients with allergic asthma and rhinitis under this therapy. IT also downmodulated the cell-surface CD66b expression *in vivo* on neutrophil from NLF. Increased MPO activity detected in the NLF of allergic patients indicates higher degree of neutrophil activation. IT significantly decreased the levels of MPO activity, neutrophil cell-surface MPO, and the number of MPO^+^CD66b^+^ cells in NLF, giving light to the molecular mechanisms involved in this therapy, which are poorly understood. Previous studies from our laboratory have shown that IT reduces MPO release after Ag-specific conjunctival challenge [15], some neutrophil functions such as the release of MPO, and reactive oxygen species [8], [33], and it has also been shown that expression of CD11b/CD18 is modulated by this therapy on human neutrophils [8], [34].

In conclusion our study shows that CD66b is upregulated by Ags which induce clinical symptoms, and that IT downmodulates the effect of Ags upon CD66b expression *in vitro*. In addition we show that IT downmodulates the levels of MPO activity and cell-surface MPO, and the number of MPO^+^CD66b^+^ cells *in vivo*. Thus, this mechanism could explain, at least in part, the effectiveness of IT. The understanding of the cells and of the cellular signalling pathways that are involved in IT not only helps us to understand the pathogenesis of allergic asthma and rhinitis, but is also important for the identification of new therapeutic targets. Thus, these observations reveal a novel mechanism with the potential to affect the adhesion of neutrophils to endothelial cells and their migration into the sites of allergic inflammation. Additional studies are required to illuminate this important question.
